# Maternal nutrient metabolism in the liver during pregnancy

**DOI:** 10.3389/fendo.2024.1295677

**Published:** 2024-03-20

**Authors:** Hongxu Fang, Qingyang Li, Haichao Wang, Ying Ren, Leying Zhang, Ling Yang

**Affiliations:** ^1^ School of Life Sciences and Food Engineering, Hebei University of Engineering, Handan, China; ^2^ College of Life Sciences, Hebei Normal University, Shijiazhuang, China

**Keywords:** glucose, hormone, lipid, liver, pregnancy, protein

## Abstract

The liver plays pivotal roles in nutrient metabolism, and correct hepatic adaptations are required in maternal nutrient metabolism during pregnancy. In this review, hepatic nutrient metabolism, including glucose metabolism, lipid and cholesterol metabolism, and protein and amino acid metabolism, is first addressed. In addition, recent progress on maternal hepatic adaptations in nutrient metabolism during pregnancy is discussed. Finally, the factors that regulate hepatic nutrient metabolism during pregnancy are highlighted, and the factors include follicle-stimulating hormone, estrogen, progesterone, insulin-like growth factor 1, prostaglandins fibroblast growth factor 21, serotonin, growth hormone, adrenocorticotropic hormone, prolactin, thyroid stimulating hormone, melatonin, adrenal hormone, leptin, glucagon-like peptide-1, insulin glucagon and thyroid hormone. Our vision is that more attention should be paid to liver nutrient metabolism during pregnancy, which will be helpful for utilizing nutrient appropriately and efficiently, and avoiding liver diseases during pregnancy.

## 1 Introduction

The liver is the largest gland of the mammalian body, and has thousands of vital functions, including efficient uptake of amino acids (AAs), carbohydrates, bile acids, cholesterol, proteins, lipids and vitamins for storage and metabolism (1). During normal pregnancy, there are essential adaptations in nutrient metabolism, which increase maternal energy reserves, in the form of glucose and lipids, to meet the maternal-fetal needs for advanced gestation (2). AA metabolism is downregulated in early and mid-pregnancy, but upregulated in late pregnancy in mice (3). Mammal nutrient supply is handled primarily by the gastrointestinal tract and the liver, and as a major metabolic hub, the liver is involved in nutrient metabolism and the synthesis of essential serum components (4). Moreover, the maternal liver systematically coordinates adaptations by activating the proneuronal transcription factor Ascl1 in the maternal hepatocytes during second half of gestation, which allows for optimal placental development and growth, and ensures the health of mother and her infant during pregnancy in mice (5). The liver controls various pathways of glucose metabolism, including glycogenesis, glycogenolysis, glycolysis and gluconeogenesis, to maintain an individual’s health by regulating several key transcription factors (6).

A successful pregnancy is dependent on correct hepatic adaptations in maternal nutrition. However, there is no systematic review that focuses on maternal liver nutrient metabolism during pregnancy. In this review, the latest information about hepatic nutrient metabolism, including glucose metabolism, lipid and cholesterol metabolism, and protein and AA metabolism, as well as maternal hepatic adaptations in nutrient metabolism during pregnancy, is discussed. In addition, the factors that regulate hepatic nutrient metabolism during pregnancy are reviewed.

## 2 Hepatic anatomy

**Figure 1:**
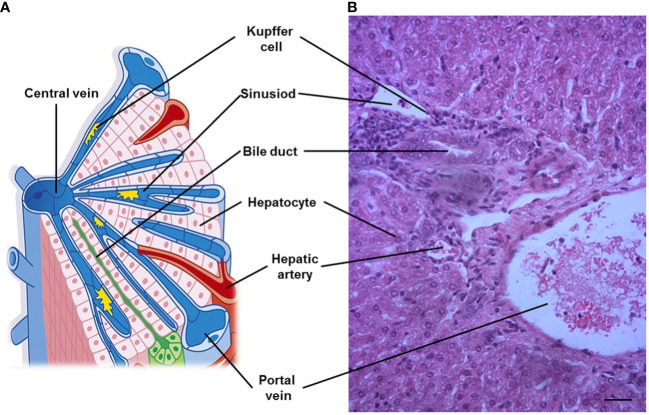
A representative hepatic lobule. **(A)** Representation of a hepatic lobule. **(B)** Hepatic tissue stained by hematoxylin and eosin. A portal triad is a component of the hepatic lobule, consists of proper hepatic artery, hepatic portal vein, small bile duct and hepatic sinusoid. Bar = 50 µm.

Despite obvious differences in hepatic lobation and gallbladder between rodents and humans, but the microscopic architecture of the liver is generally similar in all mammals, and has a critical feature for liver function. The liver is divided into lobes (the anatomical sections of the liver), and lobe is made up of hepatic lobules. Furthermore, liver cells are organized around the functional structural unit of the liver — the lobule (microscopic building blocks of the liver) (7). Branches of the portal vein and the hepatic artery merge upon and entry into the liver lobule at the portal field for blood supply of the liver, and exits at the central vein (8). In general, a typical hepatic lobule contains the portal vein, hepatic artery, bile duct and hepatic sinusoid (**Figure 1**).

## 3 Liver nutrient metabolism

### 3.1 Glucose metabolism

The liver is the major site in the body for carbohydrate biosynthesis, and plays a central role in regulating systemic glucose metabolism, and maintaining blood glucose levels within a narrow range (9). The liver regulates the balance between the uptake and storage of glucose via glycogenesis and release of glucose via glycogenolysis and gluconeogenesis to maintain blood glucose homeostasis (10). Feeding enhances insulin-mediated signaling in the liver, which shifts from a mode of net output to net uptake of hepatic glucose. This requires the activation of glycogen synthase and inhibition of glycogen phosphorylase, as well as a decline in glucagon and an increase in insulin, which lead to a decrease in hepatic glucose output from glycogen stores and gluconeogenesis in hepatocytes (6, 7).

### 3.2 Lipid and cholesterol metabolism

The liver is involved in the uptake, synthesis, packaging, and secretion of lipids and lipoproteins. The major sources of hepatic fatty acids (FAs) are dietary lipids, adipose tissue derived FAs and *de novo*-synthesized FAs (11). FA synthesis and lipid circulation occur through lipoprotein in the liver, and lipid droplets accumulate in hepatocytes (12). The liver can utilize FAs as an internal energy source through oxidative pathways, and provide energy to other organs from ketogenic products (7). Under condition of increased FA uptake, the liver often produces large amounts of the ketone bodies, including β-hydroxybutyrate, acetoacetate, and acetone, and these ketone bodies circulate among extrahepatic tissues and are metabolized (13).

Dietary triglycerides are packaged into chylomicrons within the intestinal lumen, secreted into the lymphatic system and ultimately reach the plasma, and much of the chylomicron triglycerides are taken up by muscle and adipose tissue (11). The remaining triglycerides within the chylomicron remnants are taken up by receptor mediated endocytosis to the hepatocytes, and these particles are processed by lysosomes to release FAs (14). Triacylglycerols can also be exported as constituents of very low density lipoproteins (VLDL) that are synthesized and secreted by the liver, and released into the blood (12).

Dietary cholesterol is absorbed in the intestine, and incorporated into chylomicrons that are taken up by the liver through the bloodstream, and the majority of cholesterol catabolism and excretion is also the responsibility of the liver. Approximately half of this cholesterol is excreted in the feces, and the other half is reabsorbed in the large intestine and taken up by the liver (15). The liver also plays a buffering role in regulating cholesterol homeostasis of the whole body, both by controlling several cholesterol input and elimination pathways, and serving as a storage site for the cholesterol (16).

### 3.3 Protein and amino acid metabolism

The liver is responsible for 85-90% of circulating protein synthesis, including albumin, which is essential for the maintenance of blood volume and transporting a number of critical molecules (including lipids). In addition, the liver synthesizes many AAs, glucose, and glutathione, but is also the major organ that degrades AAs. The liver breaks down proteins and metabolizes AAs to provide energy for hepatocytes, and the carbon skeleton of specific AAs is incorporated into the tricarboxylic acid cycle (7). However, only a small amount of AAs is degraded in the liver if humans and animals take up optimal amounts of AAs (17).

## 4 Maternal hepatic adaptations in nutrient metabolism during pregnancy

**Figure 2:**
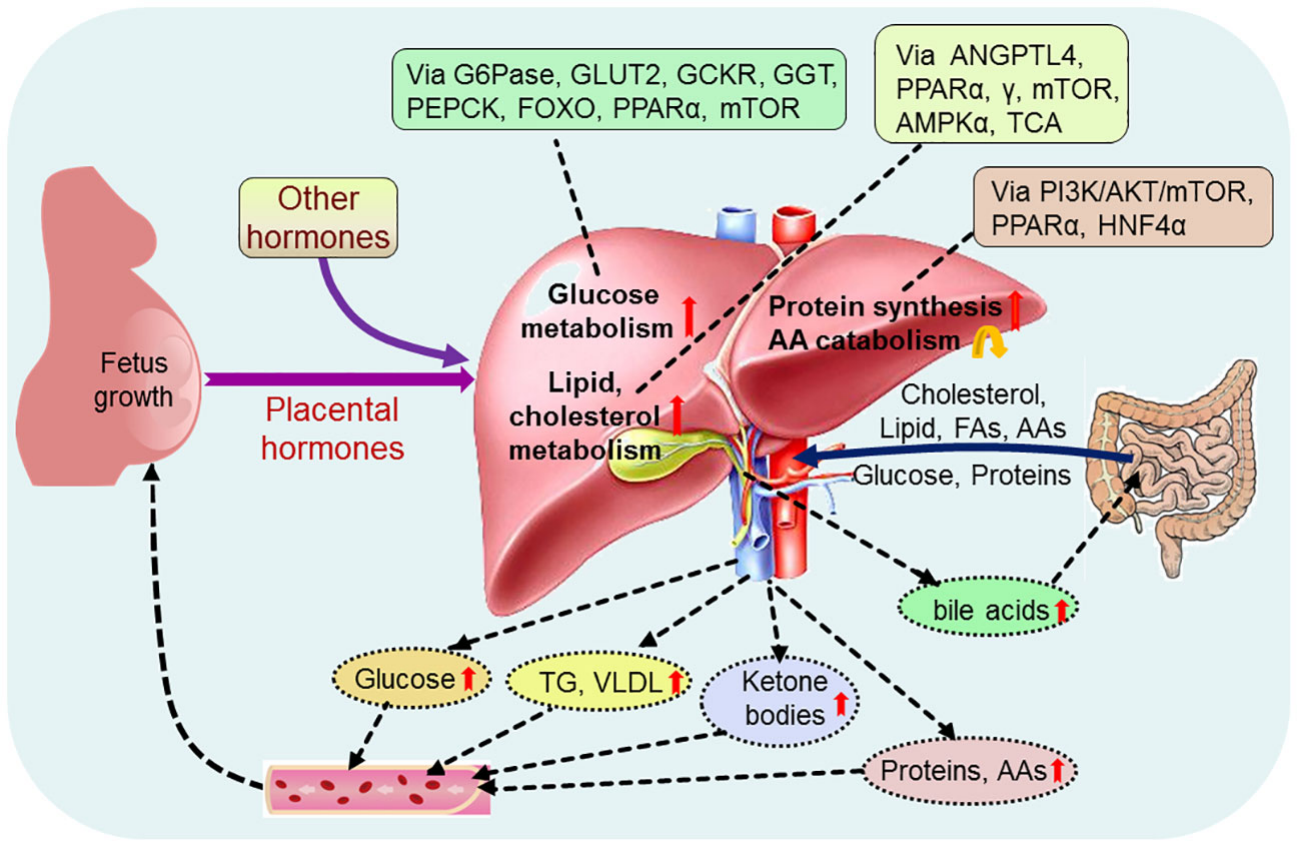
Pregnancy regulates maternal hepatic nutrient metabolism. During pregnancy, placental hormones and other hormones modulate hepatic glucose metabolism via glucose-6-phosphatase (G6Pase), glucose transporter 2 (GLUT2), glucokinase regulatory protein (GCKR), phosphoenolpyruvate carboxykinase (PEPCK), forkhead box protein O (FOXO), peroxisome proliferator-activated receptor α (PPARα) and mammalian target of rapamycin (mTOR), and regulate hepatic lipid metabolism via angiopoietin-like protein 4 (ANGPTL4), PPARα, γ, mTOR, adenosine 5’-monophosphate (AMP)-activated protein kinase α (AMPKα) and tricarboxylic acid cycle (TCA). In addition, hepatic protein metabolism, including protein synthesis and amino acid (AA) catabolism, is regulated via phosphoinositide 3-kinase (PI3K)/serine threonine kinase (AKT)/mTOR, PPARα and nuclear factor 4α (HNF4α). Glucose, cholesterol, lipid, proteins and other nutrients, including fatty acids (FAs) and amino acids (AAs), from the intestine enter into the liver, and pregnancy increases production of hepatic glucose, triglyceride (TG), very low density lipoproteins (VLDL), ketone bodies, proteins and AAs, which promote fetus growth and maternal nutrient store through the blood circulation. Furthermore, pregnancy enhances the production of hepatic bile acids, which improve lipid and glucose metabolism in the intestine.

Alterations in maternal metabolism meet the metabolic needs of the developing fetus, which is initiated very early in pregnancy. There are significant changes in hepatic nutrient metabolism in pregnant women compared with nonpregnant women, including increases in glucose metabolism, and lipid and cholesterol metabolism, protein synthesis, and a decrease AA catabolism (**Figure 2**).

### 4.1 Adaptations in hepatic glucose metabolism

**Figure 3:**
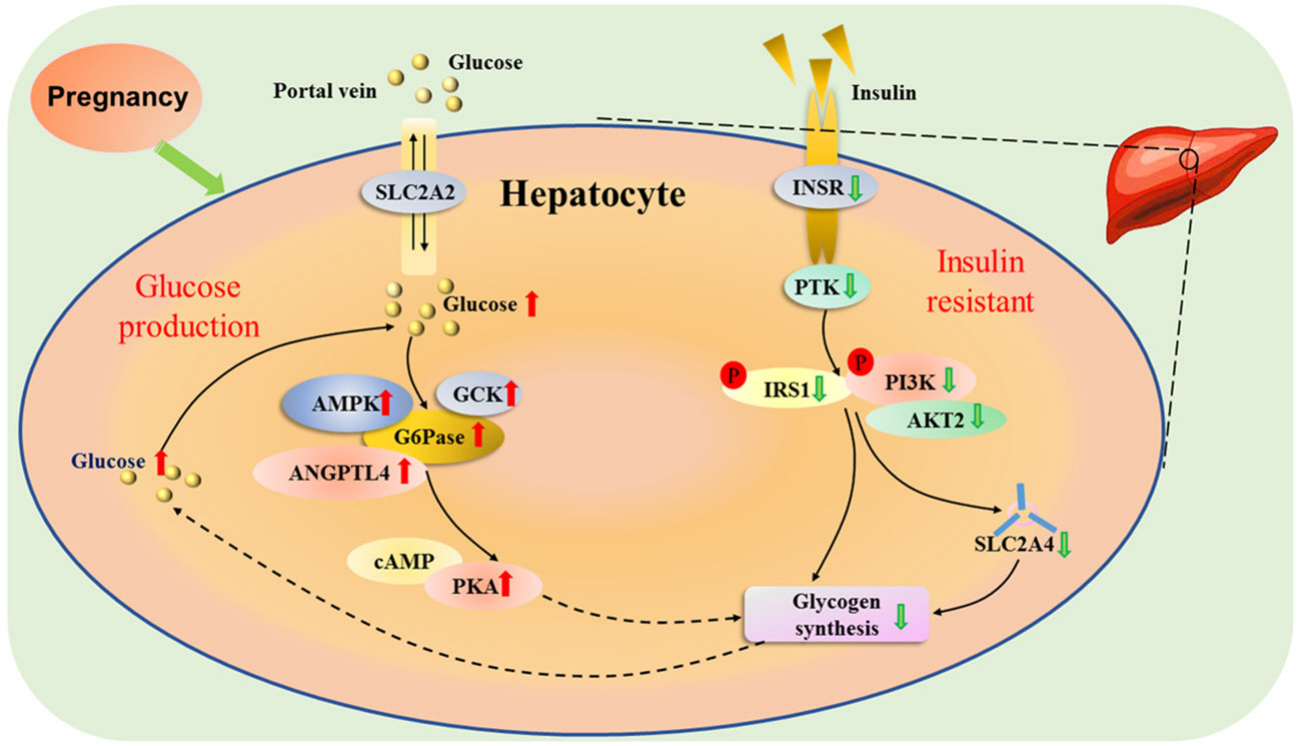
Hepatic glucose metabolic pathway during pregnancy. Pregnancy induces insulin resistance, and solute carrier family 2 member 2 (SLC2A2) mediates facilitated bidirectional glucose transport between portal vein and hepatocytes. During pregnancy, the increase in endogenous hepatic glucose production enhances the production of glucose-6-phosphatase (G6Pase), and 5’-prime-AMP-activated protein kinase (AMPK), angiopoietin like 4 (ANGPTL4) and glucokinase (GCK), which are involved in glucose homeostasis. In addition, there is an upregulation of cAMP-dependent protein kinase A (PAK), which inhibits the activity of glycogen synthesis. The glucose level increases in the liver, which reduces the activity of protein tyrosine kinase (PTK) by downregulating activity of insulin receptor (INSR). Furthermore, the phosphorylation of insulin receptor substrate 1 (IRS1) and phosphatidylinositol 3-kinase (PI3K) is inhibited, which downregulates the activity of AKT serine/threonine kinase 2 (AKT2), and reduces the ability of solute carrier family 2 member 4 (SLC2A4) transporter, resulting in a decrease in glycogen synthesis.

The appearance rates of total glucose and total gluconeogenesis are increased, which are essential for the mother to adapt to the increasing fetal demands for glucose with advancing gestation (18). In addition, adjustments in glucose production and utilization in the maternal liver are necessary for the increased glucose requirements of the gravid uterus, which depends principally on hepatic gluconeogenesis for the glucose supply, as well as hepatic glycogen synthesis during late pregnancy in ruminants (19). Furthermore, pregnancy is characterized by progressive insulin resistance, and insulin action is lower in normal late pregnancy than nonpregnant women, which results in an increase in basal endogenous hepatic glucose production during normal pregnancy (20). Maternal glucose production is enhanced from early to late pregnancy by transiently modulating maternal hepatic insulin resistance to ensure sufficient glucose delivery to the fetus (21). Pregnancy induces insulin resistance, and improves the production of glucose through inhibition of glycogen synthesis (**Figure 3**).

### 4.2 Adaptations in hepatic lipid metabolism

**Figure 4:**
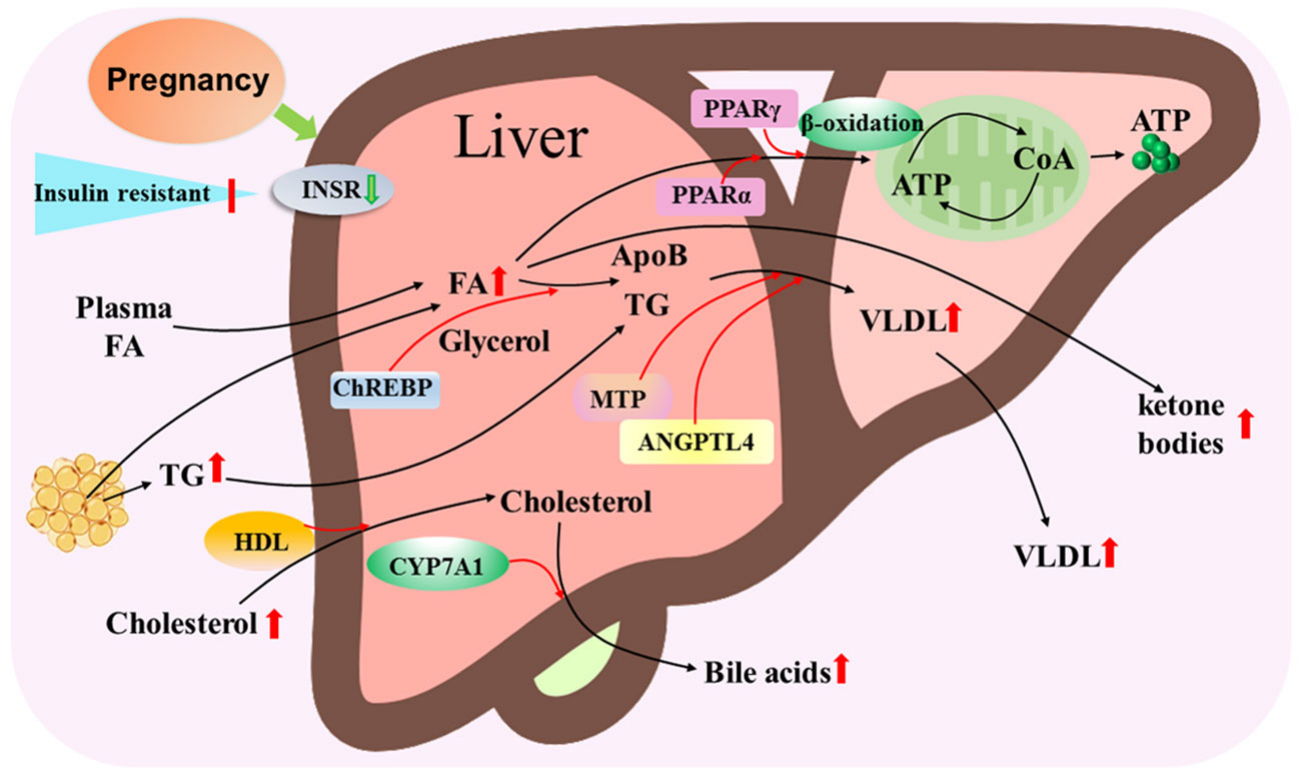
Hepatic lipid metabolic pathway during pregnancy. Pregnancy leads to insulin resistance and downregulation of insulin receptor (INSR), and increases in transport of fatty acids (FAs) from plasma and adipose tissue, triglyceride (TG) and cholesterol that are transported by high-density lipoproteins (HDL) particles to the liver. Carbohydrate-responsive element-binding protein (ChREBP) is involved in triglyceride synthesis from FAs and glycerol, and peroxisome proliferator activated receptor alpha (PPARα) and PPARγ regulate the β-oxidation of FA to release ATP through tricarboxylic acid cycle. Microsomal triglyceride transfer protein (MTP) and angiopoietin like 4 (ANGPTL4) modulate VLDL assembly by apolipoprotein B (ApoB) and TG, which result in an increase in VLDL release from the liver. In addition, cytochrome P450 family 7 subfamily A member 1 (CYP7A1) converts cholesterol to bile acids that are released from the liver to improve the digestion and absorption of lipids. Moreover, some free FAs are oxidized as ketone bodies that are released from the liver.

The mother increases the utilization of lipids as an energy source with advancing gestation, and the liver plays a central role in lipid metabolism during pregnancy. There are significant increases in serum triglycerides and total low-density lipoprotein cholesterol (LDL-C) during pregnancy in women (22). Adipose tissue, liver and placenta secrete angiopoietin-like protein 4, which inhibit lipoprotein lipase activity and are related to placental FA transfer and fetal fat accumulation during pregnancy (23). It has been reported that peroxisome proliferator-activated receptor α (PPARα) and PPARγ regulate cellular fatty acid uptake, esterification and trafficking, as well as lipoprotein metabolism (24). Pregnancy leads to an increase in the transport of FAs from plasma and adipose tissue, as well as triglyceride and cholesterol transported to the liver (**Figure 4**).

### 4.3 Adaptations in hepatic protein metabolism

Concentrations of most plasma proteins, including clotting factors, albumin, and hormone binding proteins, are changed during pregnancy. These proteins are synthesized in the liver, which are related to the physiological alterations in hepatic metabolism during pregnancy (25). Albumin synthesis actually increases in the liver during pregnancy, but lower serum concentrations are the consequence of plasma volume expansion (26). Furthermore, in humans, the level of γ-glutamyl transferase (GGT), a liver enzyme, is elevated at 10-13 weeks of pregnancy, and GGT can increase the availability of AA for intracellular glutathione synthesis and play a crucial role in defense against oxidative stress (27).

**Figure 5:**
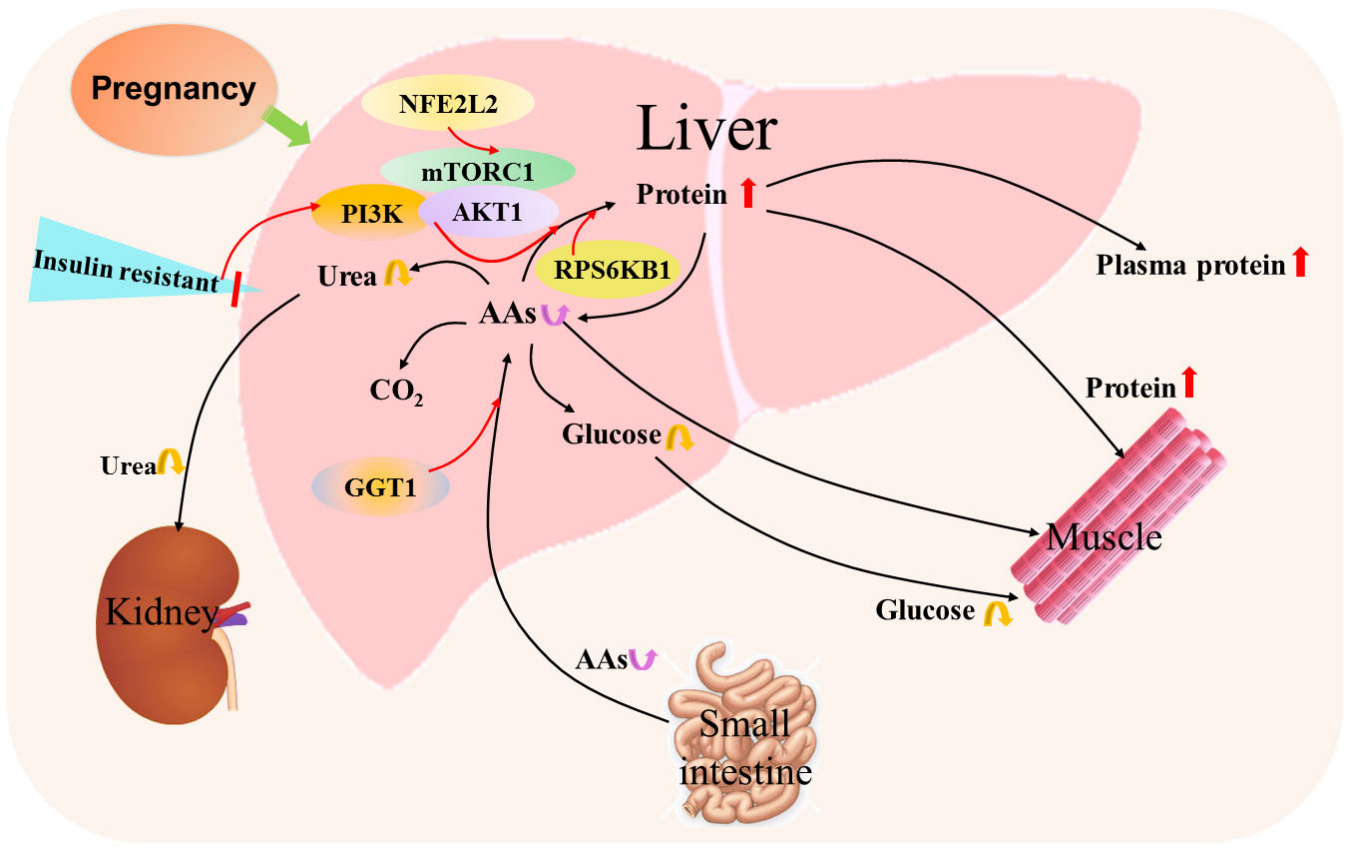
Hepatic protein and amino acid metabolic pathway during pregnancy. Pregnancy results in insulin resistance that increases protein synthesis from amino acids (AAs) by mammalian target of rapamycin (mTOR) complex 1 (mTORC1), PI3K phosphoinositide-3-kinase (PI3K) and AKT serine/threonine kinase 1 (AKT1) signaling, and ribosomal protein S6 kinase B1 (RPS6KB1) responds to mTOR signaling to promote protein synthesis. In addition, nuclear factor erythroid 2-related factor 2 (NFE2L2) is involved in the regulation of mTOR signaling. Furthermore, γ-glutamyl transferase (GGT) modulates the availability of AAs for intracellular glutathione synthesis. Moreover, the content of hepatic AAs from the small intestine through the blood circulation alters, which leads to changes in the production of glucose and urea from AAs.

Nuclear factor erythroid 2-related factor 2 is essential for regulating maternal hepatic adaptations to pregnancy by mammalian target of rapamycin signaling in mice, and modulates expression of genes encoding the denoted enzymes related to glycolysis, the pentose phosphate pathway, one carbon metabolism, nucleotide biosynthesis, glutaminolysis, fatty acid synthesis, and glutathione synthesis (28). Moreover, there are increases in the concentrations of many proteins produced by the liver, such as fibrinogen and other coagulation factors, including procoagulant factors, prothrombin fragments 1 + 2, tissue-plasminogen activator antigen and type 1 plasminogen activator inhibitor, but anticoagulants are reduced during pregnancy (29). Pregnancy results in insulin resistance, and increases protein synthesis from AAs, but a decrease in the production of glucose and urea from AAs (**Figure 5**).

## 5 Factors that regulate maternal hepatic nutrient metabolism

**Figure 6:**
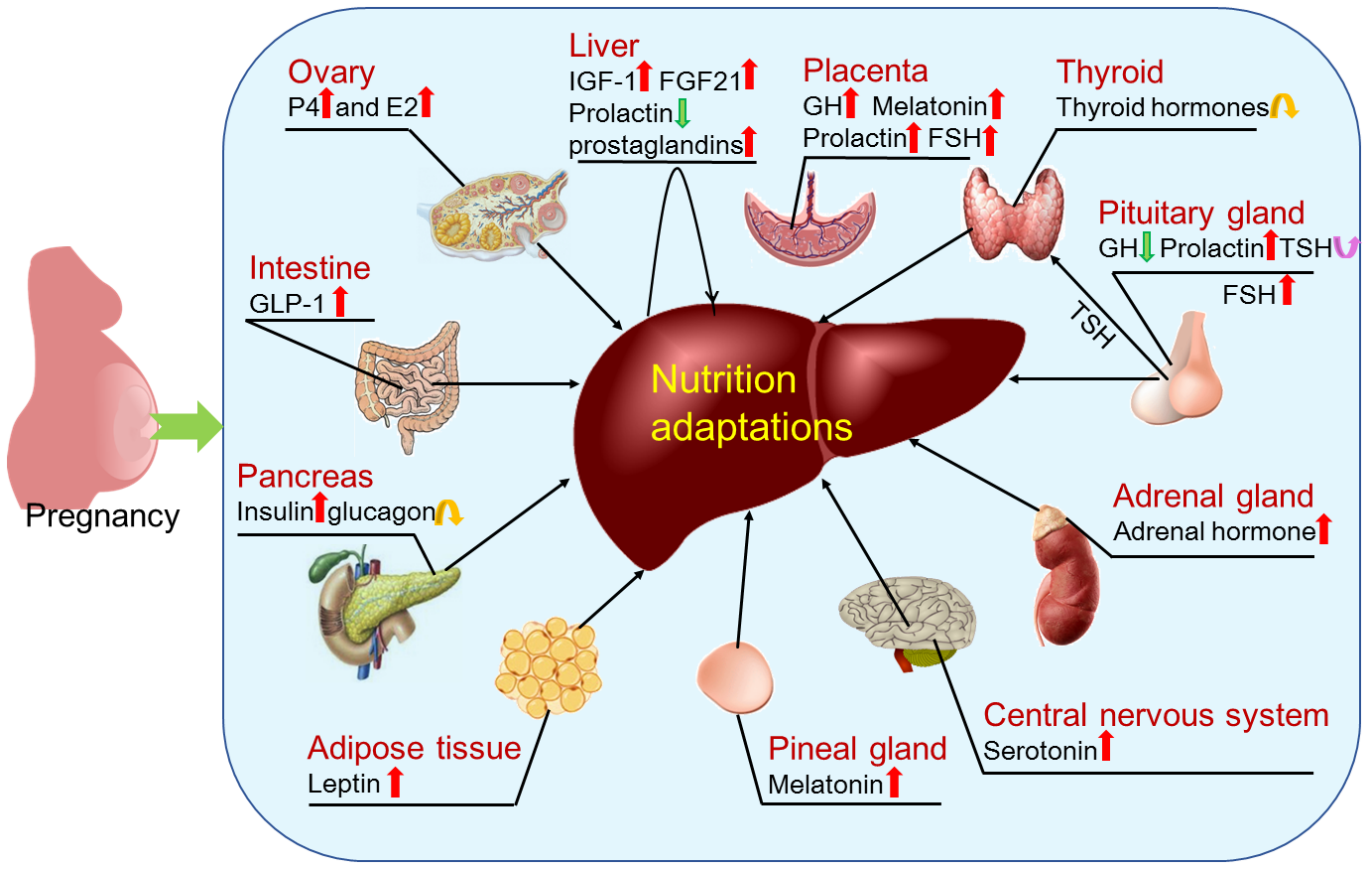
Factors that regulate hepatic adaptations in nutrient metabolism during pregnancy. During pregnancy, hepatic nutrient metabolism is modulated by factors that include estrogen (E2) and progesterone (P4) mainly produced by ovaries; insulin like growth factor 1 (IGF-1), prostaglandins and fibroblast growth factor 21 (FGF21) produced by the liver in a paracrine manner; thyroid hormones produced by thyroid; follicle-stimulating hormone (FSH), growth hormone (GH), prolactin and thyroid stimulating hormone (TSH) produced by pituitary gland; adrenal hormone mainly produced by adrenal glands; serotonin produced within the central nervous system; melatonin secreted by the pineal gland; leptin mainly synthesized in adipose tissue; insulin and glucagon secreted by pancreas; and glucagon-like peptide-1 (GLP-1) mainly released from intestinal L-cells. In addition, pregnancy regulates production of GH, melatonin, prolactin and FSH by the placenta.

Pregnancy induces maternal physiological changes by endocrine hormones and autocrine factors (30). The factors that regulate hepatic nutrition include follicle-stimulating hormone (FSH), estrogen, progesterone, growth hormone (GH)/insulin-like growth factor 1 (IGF-1), insulin, prolactin, aldosterone, adrenaline, thyroid stimulating hormone (TSH), thyroid hormone, melatonin, serotonin, glucagon and glucagon-like peptide-1 (GLP-1), leptin, prostaglandins (PGs) and fibroblast growth factor 21 (FGF21) (**Figure 6**).

### 5.1 FSH

FSH not only exert its effects on gonadal tissues, but FSH receptors (FSHRs) are also expressed in extragonadal tissues, including endothelium, monocytes, developing placenta, endometrium and fat (31). FSH can decrease body weight and modulate lipid metabolism, which are via downregulation of triglyceride concentration and PPARγ expression in the liver, and upregulation of PPARα protein level in liver and adipose tissue (32). FSH increases serum cholesterol level via inducing hepatic cholesterol biosynthesis, which is via binding to hepatic FSHRs, activating the Gi2α/β-arrestin-2/serine threonine kinase (AKT) pathway (33). FSH is related to the dysregulation of hepatic metabolism, and increased level of FSH has effects on the development of non-alcoholic fatty liver disease (34). In the placenta, vascular endothelial FSHR of fetal vessels mediates angiogenesis, and myometrial FSHR is related to the quieting of contractile activity required for successful implantation. However, the temporal upregulation of the FSHR at term pregnancy is necessary for the appropriate timing of parturition (35).

### 5.2 Estrogen

Increasing amounts of circulating estrogens suppress hepatic clearance function, which leads to increases in the levels of plasma sulfated and glucuronidated lipophilic endo- and xenobiotics. However, hepatic synthetic functions and cholesterol excretion into bile are enhanced during pregnancy (36, 37). During late pregnancy, phospholipids, cholesterol, and triglycerides increase in response to estrogen stimulation and insulin resistance, and free FAs are oxidized as ketone bodies in the maternal liver, which represent an alternative fuel source for the fetus (38). In addition, estrogen enhances liver VLDL production and decreases hepatic lipase activity during gestation in both women and experimental animals (39). Furthermore, in humans and mice, estrogen modulates liver gene expression through estrogen receptor α, β, and G-protein-coupled estrogen receptor, which is related to lipid metabolism (40).

### 5.3 Progesterone

There is an upregulation of serum progesterone levels during normal pregnancy, which suppresses hepatic clearance function, and increases hepatic synthetic functions (37). In addition, progesterone receptor is upregulated in the maternal liver during early pregnancy, which is involved in the regulation of maternal hepatic functions in an endocrine manner in an animal model (41). Progesterone reduces expression of phosphoenolpyruvate carboxykinase (PEPCK) to increase glucose uptake through transcription factor 7 like 2, a key regulator of glucose homeostasis, in liver cells (42). In addition, lipids and steroid hormones are closely linked, and steroid hormones regulate hepatic lipid production, and progesterone concentrations are related to cholesterol biosynthesis rates (43). During the latter half of pregnancy, progesterone improves the elaboration of ketones more promptly in the liver to meet the demands of advancing pregnancy (44), and there is also a rapid release of liver-synthesized triglycerides into the circulation (45).

### 5.4 Growth hormone/insulin-like growth factor 1

The pituitary gland produces GH, which modulates the production of IGF-1 via the GH receptor (GHR). GH improves body composition, fasting blood glucose levels, glucose tolerance and liver triacylglycerol levels (46), and maintains glucose metabolism through B-cell translocation gene 2 and the yin yang 1 signaling pathway in primary mouse hepatocytes and the liver (47). Hepatic GH signaling is essential for regulating intrahepatic lipid metabolism, and circulating IGF-1 can amplify the growth-promoting effects, and dampen the catabolic effects of GH (48). During pregnancy, there is an upregulation of the placental GH, which gradually replaces pituitary GH, and increases maternal IGF-1 levels (49).

IGF-1 binds to insulin receptors and stimulates insulin-like actions to regulate glucose homeostasis in the liver (50). On the other hand, IGF-1 can increase insulin resistance, and reduce triglyceride accumulation in hepatocytes (51). In addition, IGF-1 suppresses cholesterol accumulation in the liver by upregulating expression of ATP-binding cassette transporter A1 via the phosphoinositide 3-kinase (PI3K)/AKT/forkhead box protein O1 (FOXO1) signaling pathway in mice (52). IGF-1 suppresses the expression of B class scavenger receptors via PI3K/AKT pathways to participate in cholesterol metabolism in the liver (53). Moreover, IGF-1 improves VLDL assembly by upregulating mRNA abundances of apolipoprotein B (ApoB) 100, ApoE, microsomal triglyceride transfer protein, and low-density lipoprotein receptor (LDLR) in bovine hepatocytes (54).

### 5.5 Insulin

Insulin is produced by the pancreas, and as the master regulator of glucose, lipid, and protein metabolism, it can suppress hepatic glucose production and enhance hepatic glucose uptake, which results in inhibition of lipolysis and a decline in plasma free FA concentration (55). Insulin inhibits hepatic glucose production by directly acting on the liver and indirectly through its effects on the pancreas with a physiologic increase in insulin secretion (56). In addition, PPARγ coactivator 1 binds and coactivates FOXO1 to activate gluconeogenic gene expression and participate in insulin-regulated hepatic gluconeogenesis (57). Furthermore, TOX4, an insulin receptor-independent regulator of hepatic glucose production, and arrestin domain-containing 3 are involved in the modulation of insulin action and glucose metabolism in the liver (58, 59). Moreover, insulin directly controls lipid metabolism through hepatic insulin receptor and activation of AKT (60).

Insulin activates intracellular transport of lipid droplets into the smooth endoplasmic reticulum (ER) inside hepatocytes via phosphatidic acid and recruits kinesin-1, and catabolizes triglyceride-rich lipid droplets to supply FAs for producing lipoprotein particles (61). In addition, insulin regulates VLDL synthesis in hepatocytes and secretion from the liver, and FOXO1 acts on the liver to integrate hepatic insulin action on VLDL production (62). However, insulin resistance decreases catabolism of hepatic branched-chain AAs (63).

There is an increase in glucose-stimulated insulin secretion by maternal pancreatic β-cells during pregnancy (64). Therefore, insulin levels are elevated during pregnancy, which plays a vital role in promoting the uptake of glucose in insulin-sensitive tissues (the liver, muscle and fat) to ensure the growth and development of the fetus (65). However, for the obese women with gestational diabetes mellitus, insulin responses increase, but insulin sensitivity decreases with advancing gestation, which leads to an increase in basal glucose production (66).

### 5.6 Prolactin

Prolactin is primarily synthesized in the anterior pituitary gland (67), and has effects on liver gluconeogenic gene expression (68). In addition, prolactin downregulates hepatic triglyceride accumulation by downregulating stearoyl-coenzyme A desaturase 1, a rate-limiting enzyme in the biosynthesis of monounsaturated fats in female mice (69). It has been reported that early pregnancy inhibits protein expression of prolactin and prolactin receptor (PRLR) in the maternal liver of an animal model, and PRLR protein is located in the hepatocytes, endothelial cells of the proper hepatic arteries and hepatic portal veins (70). Pituitary prolactin cells increase during pregnancy, which enhance prolactin production in pituitary (71). Expression of placental lactogen increases with progression of pregnancy in the bovine placenta (72). Therefore, pregnancy regulates the expression of prolactin in the maternal liver, pituitary gland and placenta, and PRLR in the maternal liver, which has effects on hepatic triglyceride accumulation and liver gluconeogenesis.

### 5.7 Aldosterone and adrenaline

Adrenal glands mainly produce corticosteroid, aldosterone, cortisol and adrenaline type hormones. Aldosterone stimulates hepatic gluconeogenesis, and blunts the inhibitory effect of insulin (73). Furthermore, aldosterone improves the gene expression of hepatic gluconeogenic enzymes to affect the inhibitory effect of insulin on hepatic gluconeogenesis through the glucocorticoid receptor (GR) (74). On the other hand, epinephrine (adrenaline-type hormone) can promptly increase blood glucose concentration, which is mediated by a transient increase in hepatic glucose production through stimulating glycogenolysis and gluconeogenesis, but an inhibition of glucose disposal in insulin-dependent tissues (75).

In the liver, glucocorticoids (a type of corticosteroid) can inhibit the insulin receptor pathway and AKT activity, and induce FOXO1 activation via GR, which stimulates PEPCK and glucose-6-phosphatase (G6Pase) expression, hepatic glucose production and lipogenesis (76). On the other hand, upregulation of hepatic corticosterone concentration and nuclear GR activation are induced by 5α-dihydrotestosterone treatment in an animal model, which stimulates triglyceride synthesis in the liver (77). Furthermore, there is an upregulation of hepatic lipid accumulation and plasma triglyceride levels during pregnancy in mice, which occurs via upregulation of hepatic CD36 through enhancing corticosterone/cortisol levels (78).

### 5.8 Thyroid stimulating hormone and thyroid hormone

TSH is produced by the anterior pituitary, and can stimulate thyroid hormone production by the thyroid gland. TSH stimulates expression of cAMP-regulated transcriptional coactivator 2, which leads to upregulation of hepatic gluconeogenic genes and gluconeogenesis (79). In addition, TSH promotes hepatic glucose production through upregulation of G6Pase and PEPCK, and downregulation of hepatic glucokinase in the liver (80). Furthermore, TSH enhances the expression of proprotein convertase subtilisin/kexin type 9, which leads to upregulation of LDL-C and degradation of LDLR (81).

TSH regulates lipid, glucose, and energy metabolism, and modulates hepatic bile acid homeostasis via a sterol regulatory element binding protein-1c (SREBP)-2/HNF-4α/cholesterol 7α-hydroxylase signaling pathway independent of thyroid hormones (82). Furthermore, thyroxine treatment enhances the relative rate of triacylglycerol synthesis from glycerol, and downregulates the accumulation of diacylglycerol in rat liver (83). Moreover, thyroid hormone plays essential roles in hepatic lipid synthesis and FA oxidation, which is dependent on the transcription factor carbohydrate-responsive element-binding protein in hepatocytes (84). There is a transient increase in free thyroxine levels, but a decrease in TSH concentrations during the first trimester. However, free thyroxine concentrations decrease approximately 10 to 15%, and serum TSH values return to normal after the first trimester (85).

### 5.9 Melatonin

The pineal gland secretes melatonin that acts directly on the liver to elevate the plasma glucose level via melatonin receptor 1B in mouse hepatocytes in a dose-dependent manner (86). Maternal melatonin and placental melatonin levels increase progressively until term during normal pregnancy (87). In addition, melatonin treatment inhibits glucose uptake and ATP production via downregulation of glucose transporter 3 and Yes-associated protein that are key regulators of Hippo signaling pathway in hepatocellular carcinoma cells (86). Furthermore, melatonin treatment improves hepatic insulin resistance and steatosis (88), attenuates lipid accumulation, and enhances the activity of AMP-activated protein kinase (AMPK) mediated by the melatonin receptor 1A signaling pathway in the liver of rats (89). Moreover, melatonin affects lipolysis by activating phosphorylation of AMPK, inactivating acetyl-CoA carboxylase, upregulating PPARα, but downregulates SREBP-1c, FA synthase, and stearoyl-CoA desaturase-1 in HepG2 cells (90). It has been reported that melatonin receptor 1A is upregulated in the maternal liver, but melatonin receptor 1B is downregulated during early pregnancy in an animal model (91), which may affect lipid and glucose metabolism during pregnancy.

### 5.10 Serotonin

Serotonin (5-hydroxytryptamine) produced within the central nervous system promotes energy expenditure via sympathetic drive, and is also secreted by peripheral tissues. Serotonin enters the bloodstream to promote the body for energy storage in the liver (92). In addition, serotonin functions as a hormone in central and peripheral systems to regulate systemic energy homeostasis, and participates in hepatic metabolism via its receptors (68). Furthermore, supplementation with 5-hydroxytryptophan improves expression of hepatic serotonin receptors, and glycolytic and gluconeogenic enzymes in dairy calves (93). Moreover, serotonin injection increases hepatic glycogen synthesis and concentrations, as well as hepatic cholesterol content, and stimulates the contraction of the gallbladder and excretion of bile (94). The concentration of hepatic serotonin, glucose transporters and expression of serotonin receptor are dynamic in the liver, suggesting that serotonin is essential for liver glucose homeostasis via its receptor in the liver during the transition from pregnancy to lactation in an animal model (95). There is an increase in serotonin expression in islets during pregnancy, which enhances glucose-stimulated insulin secretion, and contributes to maintaining glucose homeostasis and sensitivity in the liver (64).

### 5.11 Glucagon and glucagon-like peptide-1

Glucagon is a pancreas-derived hormone that exerts its function by binding to glucagon receptor (GCGR) that is mainly expressed in the hepatic periportal area (96). Glucagon enhances lipid oxidation and VLDL assembly via GCGR, and downregulates lipid synthesis in the hepatocytes, which stimulates the transportation of intracellular triglycerides, and downregulates liver fat accumulation in an animal model (97). In addition, glucagon can induce expression of gluconeogenic genes (G6Pase and PEPCK) through cAMP-response element-binding protein, and trigger a second delayed phase of FA oxidation gene expression to improve hepatic lipid homeostasis in mice (98). Glucagon-induced acetylation of cyclic AMP-responsive element binding protein, hepatocyte specific (CREBH) and SREBP-c1 suppresses hepatic lipid synthesis, and glucagon-induced acetylation of PPAR-α and FOXa2 enhances hepatic FA oxidation (99). Plasma glucagon level increases significantly between the 16th and the 28th week of gestation, but returns to normal at the last trimester of pregnancy in women (100).

GLP-1 is mainly released from intestinal L-cells in response to meal ingestion, and active GLP-1 can reach the liver through the circulation (101). GLP-1 receptor is located within the vicinity of the entrance of the hepatic portal vein, which is critically associated with portal glucose sensing (102). GLP-1 can directly stimulate insulin secretion and inhibit glucagon release, and has direct effects on increasing the activity of glycogen synthase α, decreasing the activity of glycogen phosphorylase α to promote the incorporation of glucose into glycogen in hepatocytes independent of insulin and glucagon (103). In addition, GLP-1 analogs reduce hepatic endogenous glucose production, and *de novo* lipogenesis, but increase hepatic insulin sensitivity (104). Furthermore, GLP-1 analogs bind to their receptors to enhance hepatic insulin sensitivity, and modulate gene expression involved in FA oxidation and *de novo* lipogenesis in the liver (105). There is an increase in the production of islet-derived GLP-1 during pregnancy (106).

### 5.12 Leptin

Leptin is mainly synthesized in white adipose tissue, and secreted from the placenta during pregnancy, which plays an important role in the maintenance of maternal and fetal glucose metabolism (107). Leptin decreases blood glucose level, and modulates glucose and lipid metabolism in the liver (108). In addition, leptin enhances hepatic acetyl-coenzyme A carboxylase phosphorylation, FA oxidation and ketogenesis (109), and increases microsomal triglyceride transfer protein expression in hepatic cells via leptin receptors, which are involved in lipid transportation from the liver to peripheral tissues (110). Furthermore, hepatic Kupffer cells facilitate the effects of leptin on upregulation of hepatic FA oxidation and downregulation of triglycerides dependent on PI3K activity via leptin receptor in the liver (111).

Maternal obesity during pregnancy increases the risk of offspring developing obesity, and obesity is characterized by elevated levels of leptin in pregnant females (112). A state of leptin resistance in the liver during mid-pregnancy helps maintaining lipogenic metabolism, but an opposite pattern in late pregnancy favors catabolic metabolism in the liver, which is independent of progesterone and prolactin (113). Therefore, the mechanism by which leptin regulates maternal hepatic lipid metabolism requires investigation in the future.

### 5.13 Prostaglandins

PGD2 can regulate glucose homoeostasis and/or other specific metabolic processes inside parenchymal liver cells (114). In addition, PGs mediate intercellular communication between liver cell populations in regulating liver carbohydrate metabolism, and PGF2α, PGD2 or PGE2 treatment increases glucose output (115). Furthermore, PGs, mainly PGD2, from Kupffer and endothelial cells can influence glucose release from hepatic parenchymal cells (116), and PGE2 can attenuate fat deposition in mouse primary hepatocytes (117). In addition, cyclooxygenase-2, PGE synthase and PGF synthase are upregulated in the maternal liver during early pregnancy, which are related to maternal hepatic function adjustment during early pregnancy in an animal model (118).

### 5.14 Fibroblast growth factor 21

FGF21 is mainly secreted by the liver, and modulated by glucosamine to improve hepatic glucose metabolism via the nuclear factor kappa B, p38 and protein kinas pathways (119). In addition, Th2 cytokines interleukin-4 (IL-4) and IL-13 enhance expression of FGF21 to modulate energy metabolism via the IL-4/IL-13-signal transducer and activator of transcription 6 axis in the liver (120). Furthermore, adiponectin couples FGF21 actions to mediate hepatic glucose homeostasis and insulin sensitivity, which attenuates hepatic steatosis and obesity-induced impairment in mice (121). Moreover, exenatide and liraglutide induce hepatic FGF21 synthesis, which suppresses the activities of G6Pase and PEPCK induced in a paracrine manner in hepatocytes, and controls energy homeostasis in an endocrine manner (122).

Hepatic FGF21 is a critical regulator of lipid homeostasis, which is modulated by PPARα to change expression of key genes for governing lipid and ketone metabolism in the liver (123). In addition, FGF21 can directly regulate lipid metabolism and reduce lipid accumulation to reverse nonalcoholic fatty liver disease through an insulin-independent pathway, which is associated with retinoid-related orphan receptor response element, PPARγ coactivator-1α, and retinoid acid receptor-related orphan receptor α in hepatocytes (124). Furthermore, expression of FGF21 gene is increased in the liver of dairy cows during the transition from pregnancy to lactation (125), and the liver is the major source of plasma FGF21 in late pregnancy (126). Moreover, there is increased FGF21 secretion and hepatic triglyceride content during pregnancy, indicating that FGF21 is involved in balancing lipid homeostasis and meeting maternal and infant energy requirements in late pregnancy (127).

## 6 Conclusions

It is a worldwide problem that half of pregnant women suffer from maternal obesity and other pregnancy-associated liver diseases (128). It is reviewed in this paper that pregnancy induces insulin resistance, and improves the production of glucose, increases the transport of FAs, triglyceride and cholesterol, protein synthesis from AAs, but decreases the production of glucose and urea from AAs. In addition, some endocrine hormones and autocrine factors have effects on glucose metabolism, lipid and cholesterol metabolism, and protein metabolism. Therefore, more studies are necessary to determine food-based dietary guidelines for the pregnant women and the regulatory mechanisms of maternal hepatic nutrition adaptations depending on the pregnancy stages. Furthermore, success in these areas will bring new hopes for pregnancy precision nutrition and prevention of pregnancy-related nutrition diseases.

## Author contributions

HF: Investigation, Writing – original draft. QL: Investigation, Writing – original draft. HW: Writing – review & editing, Validation, Software. YR: Writing – review & editing, Validation, Software. LZ: Writing – review & editing, Supervision, Conceptualization. LY: Writing – review & editing, Supervision, Conceptualization.
